# Intraoperative renal and cerebral tissue oxygen saturation measurements to predict postoperative acute kidney injury in pediatric cardiac surgery: a prospective observational study

**DOI:** 10.1007/s10877-025-01345-4

**Published:** 2025-09-03

**Authors:** Dario Massari, Marco Modestini, Cornelia K. Niezen, Lu Yeh, Anna Carina Zoutman, Thomas W. L. Scheeren, Ryan E. Accord, Kai van Amsterdam, Michel M. R. F. Struys, Jaap Jan Vos

**Affiliations:** 1 Department of Anesthesiology, University of Groningen, University Medical Center Groningen, PO Box 30.001, Hanzeplein 1, 9700 RB Groningen, The Netherlands; 2 Department of Anesthesiology, Isala Hospital, Zwolle, The Netherlands; 3 BD Advanced Patient Monitoring, Heidelberg, Germany; 4 Department of Cardiothoracic Surgery, University of Groningen, University Medical Center Groningen, Groningen, The Netherlands; 5 Department of Basic and Applied Medical Sciences, Ghent University, Gent, Belgium

**Keywords:** Acute kidney injury, Near-infrared spectroscopy, Cerebral oxygenation, Renal oxygenation, Tissue oxygenation, Congenital heart disease

## Abstract

**Purpose:**

Pediatric patients undergoing cardiac surgery are at risk of developing postoperative acute kidney injury (AKI). We hypothesized that a reduction in intraoperative renal (SrO_2_) or cerebral (ScO_2_) tissue oxygen saturation is associated with postoperative AKI.

**Methods:**

We conducted a prospective observational study including fifty pediatric patients with non-cyanotic heart disease undergoing elective surgical repair with cardiopulmonary bypass. Intraoperative SrO_2_ and ScO_2_ were monitored using near-infrared spectroscopy (O3^®^ Regional Oximetry). Relative decreases of 10% and 20% from baseline SrO_2_ and ScO_2_ were analysed, calculating the total time below the threshold, area under the threshold, and time-weighted average. The primary outcome was the association between intraoperative SrO_2_ and ScO_2_ decreases, and the occurrence of postoperative AKI defined with the ‘Kidney Disease: Improving Global Outcomes’ criteria. Secondary outcomes included the association between other known or potential risk factors for AKI and postoperative AKI.

**Results:**

The incidence of postoperative AKI was 18.4%. There was no association between the duration and extent of intraoperative reductions of SrO_2_ and ScO_2_ below 10% and 20% from baseline, and postoperative AKI (e.g., area under the threshold for ScO_2_ decreases below 10%: 36.8 [11.8 to 419.9] % min in patients with AKI vs. 9.6 [0.6 to 92.8] % min in patients without AKI, *P* = 0.117). Preoperative serum creatinine, body mass index, intraoperative hypotension, and blood lactate were associated with postoperative AKI.

**Conclusion:**

A decrease in intraoperative renal or cerebral tissue oxygen saturation was not associated with postoperative AKI in pediatric patients undergoing surgery for non-cyanotic congenital heart disease.

**Supplementary Information:**

The online version contains supplementary material available at 10.1007/s10877-025-01345-4.

## Introduction

Postoperative acute kidney injury (AKI) is a frequent complication after cardiac surgery in pediatric patients, ranging from 20 to 86%, depending on the underlying congenital heart defect and on the criteria used to define AKI [1–4]. Morbidity and mortality are higher in pediatric patients who develop postoperative AKI, which still stands as a relevant clinical challenge in cardiac surgery [5–7].

Established risk factors for AKI after pediatric cardiac surgery include younger age, lower body weight, lower preoperative serum creatinine, longer cardiopulmonary bypass (CPB) time, and vasopressor use [5]. Intraoperative hypotension is associated with postoperative AKI in adults [8], while its role in pediatric patients is less clear [9]. One key pathogenetic mechanism involved in the development of AKI is perioperative renal hypoperfusion and hypoxia. Near-infrared spectroscopy (NIRS), a non-invasive monitoring technique that allows the real-time assessment of regional tissue oxygenation, has been used to directly measure renal tissue oxygen saturation (SrO_2_) in children [10–12], despite limited evidence and validation for this technique [11]. Cerebral tissue oxygen saturation (ScO_2_) monitoring is a common practice during anesthesia for pediatric cardiac surgery, not only as a direct assessment of cerebral perfusion and oxygenation, but also because ScO_2_ can reflect the microvascular function of other tissues and organs, including the kidney [13].

There is conflicting evidence regarding the relationship between perioperative SrO_2_ and ScO_2_ values and the occurrence of postoperative AKI in pediatric patients, with some studies showing a relationship between decreases in SrO_2_ [14–16] or ScO_2_ [17] values and the development of AKI. Limitations of these studies included the enrolment of heterogeneous cohorts of patients – e.g. with both cyanotic and non-cyanotic congenital heart defects [2, 14, 18, 19] – and the use of different definitions of AKI.

Since the evidence derived from studies conducted in pediatric cardiac surgery is inconclusive, it remains unclear whether monitoring renal or cerebral tissue oxygen saturation is a clinically applicable tool for predicting postoperative AKI in this population. We designed this study to hypothesize that a decrease in intraoperative SrO_2_ and ScO_2_ values is associated with the development of postoperative AKI in pediatric patients undergoing corrective surgery for non-cyanotic congenital heart disease with a left-to-right shunt [20]. Secondarily, we investigated the association between other known or potential predictors of AKI (age, body mass index, preoperative serum creatinine, CPB time, intraoperative hypotension, vasopressors and inotropes infusion, red blood cell transfusion, fluid balance, and blood lactate) and postoperative AKI.

## Methods

This prospective observational study was conducted at the University Medical Center Groningen, the Netherlands, from 2021 to 2024. The Medical Ethics Committee of the University Medical Center Groningen (Chairperson Prof. W.A. Kamps) reviewed our research protocol on April 20th, 2021 (METc 2021/191) and determined that it did not fall under the Medical Research Involving Human Subjects Act (WMO). The study was successfully registered in the Netherlands Trial Register on November 3rd, 2021 (NL9852). The study results are reported in accordance with the ‘Strengthening the Reporting of Observational Studies in Epidemiology’ (STROBE) guidelines [21]. Written informed consent was obtained from both parents before surgery.

### Study population

We included patients under eighteen years of age with a non-cyanotic congenital heart defect with a left-to-right shunt scheduled for corrective cardiac surgery with CPB [20]. The exclusion criteria were a cerebral disease, renal disease or preoperative serum creatinine > 100 µmol l^−1^ (1.13 mg dl^−1^), structural renal abnormalities, extreme prematurity (gestational age < 32 weeks), limitations in the positioning of NIRS sensors (e.g. skin defects or diathermia pad placement), and known or suspected allergies to the glue of the NIRS sensors. Since data about AKI incidence for this patient population in our center were not available, we calculated the expected AKI incidence based on literature data. Previous studies conducted in pediatric cardiac surgery and using the KDIGO criteria found an AKI incidence ranging from 35 to 86% [2, 22–24]. These studies included both cyanotic and non-cyanotic patients. Since cyanotic disease is a known risk factor for AKI [5, 25] we expected the AKI incidence in non-cyanotic patients to be on the lower side; therefore, we assumed a 30% AKI incidence to calculate the sample size. To obtain an Area Under the Receiver Operating Characteristic curve (AUROC) of 0.75 for predicting AKI [15, 26], with 80% power and a two-tailed 95% confidence level, a sample size of 46 patients was required. This was increased to 50 to compensate for possible missing data.

### Anesthetic management

Before the induction of anesthesia, standard non-invasive monitoring was instituted. General anesthesia was induced by sevoflurane inhalation. After orotracheal intubation, mechanical ventilator settings were adjusted based on the patient’s weight and peripheral oxygen saturation. The fraction of inspired oxygen (FiO_2_) was set at 0.40 as a default, but the attending anesthetist was free to increase it according to clinical needs. A radial artery cannula was placed to measure invasive continuous blood pressure, and a central venous line was inserted into the internal jugular vein under ultrasound guidance. Internal and peripheral body temperatures were continuously monitored, and temperature management was guaranteed by convective air warming. Patients who underwent correction of isolated atrial septal defect were kept normothermic. Patients who underwent correction of a ventricular septal defect, an atrioventricular septal defect, or combined defects had a target temperature of 34 °C. Two O3^®^ Regional Oximetry sensors connected to a Root^®^ oximetry monitor (Masimo Corporation, Irvine, California, USA) were placed bilaterally on the forehead for ScO_2_ monitoring (Infant and Neonatal Adhesive Sensor for patients < 10 kg, or Pediatric Adhesive Sensor for patients > 5 kg and < 40 kg, as per manufacturer specifications). Two additional sensors (of the same size as the forehead sensors) were placed bilaterally on the flanks for SrO_2_ monitoring: ultrasound guidance was used to locate the kidneys, and the O3 NIRS sensors were placed on the overlying skin region. The depth of the kidney capsule beneath the skin surface was recorded. NIRS sensors were placed by a dedicated researcher. SrO_2_ and ScO_2_ baseline values were recorded during the five minutes before incision after obtaining stable NIRS measurements at a constant (i.e., stable for at least 5 min) FiO_2_. Since intraoperative ScO_2_ monitoring is a standard of care in our center for pediatric patients undergoing cardiac surgery, the anesthetist was not blinded to the tissue oxygen saturation values. According to our standard practice, when ScO_2_ drops more than 20% from its baseline value or falls below a 50% absolute value, interventions are taken to restore normal ScO_2_ values (e.g., adjusting ventilation, administering blood products, or adjusting the CPB pump flow) [27].

### Surgical technique

Surgery was performed through a median sternotomy by two experienced pediatric cardiothoracic surgeons. Arterial cannulation for CPB was performed in the aortic arch, and venous drainage was performed using bicaval cannulation. The cardioplegic solution (Custodiol 20–40 ml kg^−1^) was administered using the antegrade technique.

### Data registration

All respiratory and hemodynamic variables, SrO_2_, and ScO_2_ values were measured continuously and automatically recorded in our electronic patient high-frequency measurements database every second to four seconds. The attending anesthetist manually registered the drugs and fluids administered, transfusions of blood components, duration of surgery, CPB, and aortic clamp time in the electronic patient database (EPIC, Verona, Wisconsin, USA). The attending perfusionist recorded all solutions administered via the CPB circuit, hematocrit levels during CPB, and oxygenation levels in both the premembrane and postmembrane oxygenators, as well as ventilation values.

### Outcomes

Postoperative AKI occurring within the third postoperative day was defined according to the ‘Kidney Disease: Improving Global Outcomes’ (KDIGO) criteria [28, 29]. Preoperative serum creatinine was measured the day before surgery. Postoperative serum creatinine was measured at intensive care unit (ICU) admission, once daily until the patient was discharged from the ICU (or at least until postoperative day two if ICU discharge occurred earlier), and subsequently as clinically necessary. Urine output was recorded daily as long as the urinary catheter was in place.

The primary outcome of the study was the association between intraoperative decreases in SrO_2_ and ScO_2_ values, and postoperative AKI. Secondary outcomes included the association between other known or potential predictors of AKI (age, body mass index (BMI), preoperative serum creatinine, CPB time, intraoperative hypotension, vasopressors and inotropes infusion, red blood cell transfusion, fluid balance, blood lactate), and postoperative AKI. Finally, a post-hoc exploratory analysis was conducted to assess whether a reduced intraoperative SrO_2_-ScO_2_ gradient (recently shown to be associated with hypoperfusion during CPB) [30] was associated with postoperative AKI.

### Statistical analysis

Continuous data were tested for normality using the Shapiro-Wilk test. Non-parametric data are presented as median [interquartile range], and parametric data are presented as mean ± standard deviation. Differences in clinical variables between patients with and without postoperative AKI were tested using the Mann–Whitney U test, Student’s t-test, or Fisher’s exact test as appropriate. The linear correlation between continuous variables was assessed using the Pearson’s correlation coefficient (*r*).

**Fig. 1:**
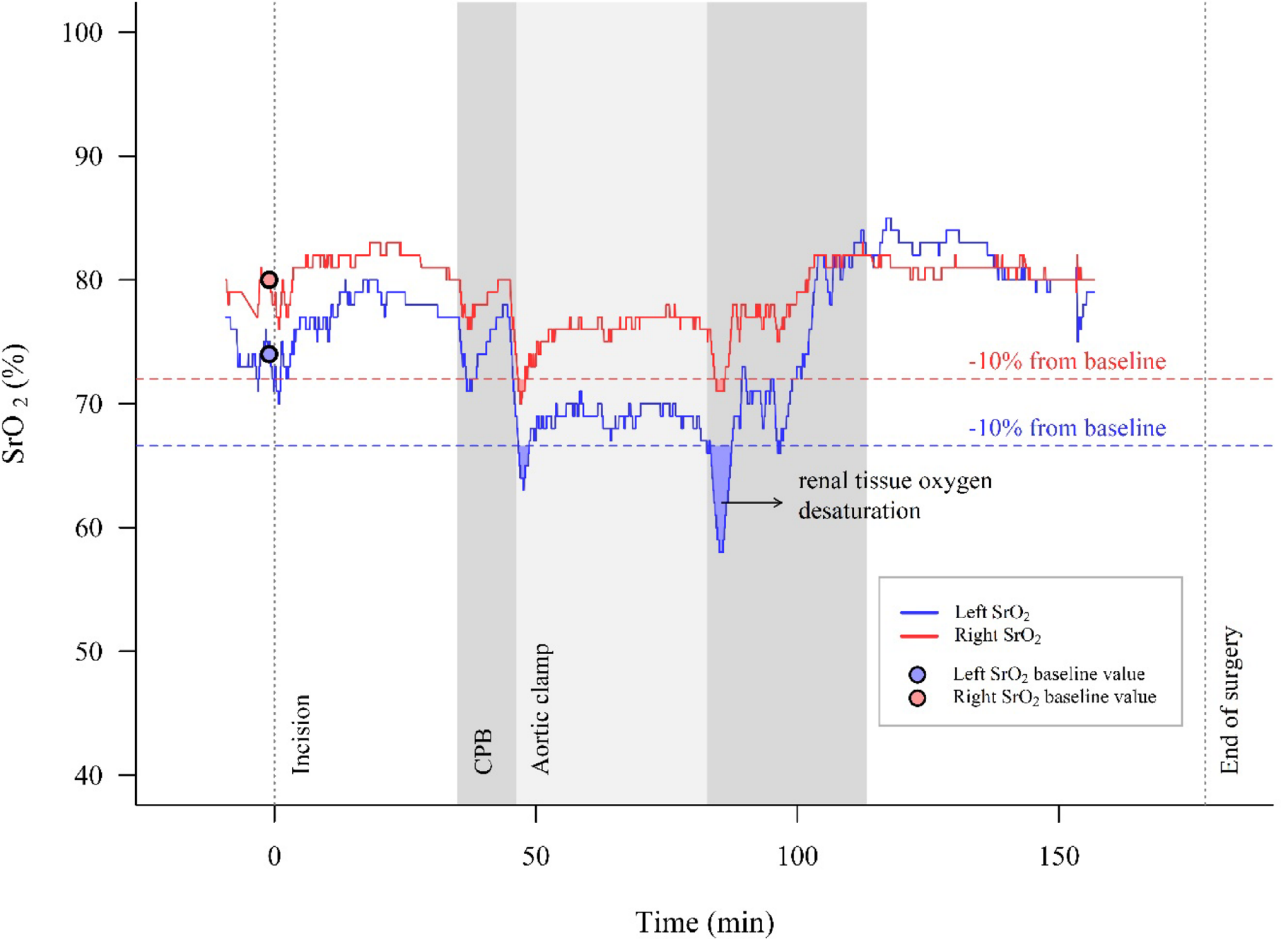
Intraoperative renal tissue oxygen saturation. Intraoperative recording of left (blue) and right (red) renal tissue oxygen saturation (SrO_2_) for a single patient. The horizontal dashed lines represent a relative 10% decrease from the respective baseline SrO_2_ value (blue and red dots). The red and blue shaded areas represent the area under the threshold (AUT). The total time below the threshold, the AUT, and the time-weighted average were calculated separately for left and right near-infrared spectroscopy sensors and then averaged for subsequent analysis. The grey and light-grey shaded areas represent the cardiopulmonary bypass and aortic clamp time, respectively

The analysis of intraoperative reductions in SrO_2_ and ScO_2_ values was performed by a researcher blinded to the occurrence of postoperative AKI. We considered two different tissue oxygen saturation thresholds for both variables: a relative decrease below 20% from the respective baseline value, which has been used as a threshold in previous studies [16, 31, 32], and a relative decrease below 10% from baseline to investigate milder degrees of tissue hypoxia (Fig. 1). For both SrO_2_ and ScO_2_, we calculated the total intraoperative time below the threshold, the area under the threshold (AUT), and the time-weighted average (TWA, computed by dividing the AUT by the duration of surgery). Since we placed two cerebral and two flank NIRS sensors, we first calculated two separate values for the left and right NIRS sensors. The two values were then averaged, and the resulting average value was used for subsequent analysis. Receiver Operating Characteristic (ROC) curves were used to assess the performance of intraoperative SrO_2_ and ScO_2_ decreases in predicting postoperative AKI, and the AUROC was calculated. For the post-hoc exploratory analysis, we calculated the cumulative intraoperative SrO_2_-ScO_2_ gradient as the area between the SrO_2_ and ScO_2_ curves [30], and the average intraoperative gradient by dividing the obtained area by the duration of surgery.

Intraoperative hypotension was defined as a decrease below the 5th percentile of mean arterial pressure (MAP) for age – which has previously been used as a definition of hypotension in hospitalized pediatric patients [33] – and the total intraoperative time below the threshold, AUT, and TWA were computed as described above. The use of vasopressors and inotropes was quantified by calculating the vasoactive-inotropic score [34].

Finally, the relationship between known or potential predictors of AKI and postoperative AKI was assessed using a logistic regression model. Univariate and multivariate analyses were performed. In the multivariate analysis, backward elimination was applied to identify the final model based on the lowest Akaike information criterion, and goodness of fit was assessed using McFadden’s pseudo-R-squared. Adjusted odds ratios (aORs) and their 95% confidence intervals (95% CI) were computed. Multicollinearity between predictor variables was tested by calculating the variance inflation factor.

Linear interpolation for SrO_2_, ScO_2_ and MAP values was used for intraoperative sections of missing data shorter than 5 min. If the section was longer than 5 min, the entire data series was omitted from the analysis. No inference was made for other missing data. Statistical analysis was performed using R version 4 (R Core Team, Vienna, Austria, 2020). Statistical significance was set at a two-tailed *P* value < 0.05 for all tests.

## Results

**Fig. 2:**
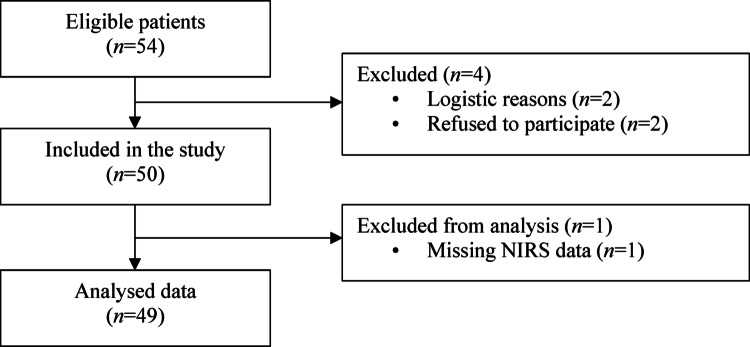
Flow diagram of the study. NIRS: near-infrared spectroscopy

Fifty consecutive patients were enrolled from October 27th, 2021, to January 23rd, 2024. One patient was excluded from the analysis because of missing SrO_2_ and ScO_2_ values owing to technical issues. The final analysis was conducted on forty-nine patients (Fig. 2). Patients underwent surgical repair of ventricular septal defect (*n* = 20), atrial septal defect (*n* = 16), atrioventricular septal defect (*n* = 9), combined atrial and ventricular septal defects (*n* = 3), or aortopulmonary window (*n* = 1). Patient characteristics are reported in Table 1. The kidney depth beneath the skin surface ranged from 0.8 to 3.8 cm, being ≤ 2 cm in forty patients, 2 to 2.5 cm in eight patients, and > 2.5 cm in one patient. Kidney depth had a weak linear correlation with BMI (*r* = 0.37, *P* = 0.009) but not with age (*r* = 0.18, *P* = 0.229). Baseline SrO_2_ values did not correlate with BMI or kidney depth. A positive correlation was found between baseline partial pressure of arterial oxygen (PaO_2_) and both baseline SrO_2_ (*r* = 0.32, *P* = 0.026) and ScO_2_ (*r* = 0.32, *P* = 0.025). Intraoperative FiO_2_ data are reported in Table 1 and Online Resource 1.

**Table 1:** Patient characteristics, preoperative, intraoperative and postoperative clinical variables

Patient characteristics	All patients (*n* = 49)	No AKI (*n* = 40)	AKI (*n* = 9)	*P* value
Age (months)	7.4 [5.7 to 29]	8.4 [5.6 to 31.1]	6.2 [6 to 7.7]	0.320
Sex, female (*n*)	21 (43%)	16 (40%)	5 (56%)	0.470
Weight (kg)	7.4 [6.1 to 12.4]	7.8 [6.2 to 14.1]	6.1 [5.9 to 8.4]	0.121
Length (cm)	67 [62 to 90]	69 [62 to 93]	65 [62 to 68]	0.409
BMI (kg m^−2^)	15.6 ± 1.6	16 ± 1.4	15.2 ± 2.3	0.346
BSA (m^2^)	0.37 [0.32 to 0.54]	0.38 [0.33 to 0.60]	0.32 [0.32 to 0.40]	0.159
Kidney depth (cm)	1.7 [1.2 to 1.9]	1.7 [1.1 to 1.9]	1.8 [1.7 to 1.9]	0.437
**Preoperative variables**				
Serum creatinine (mg dl^−1^)	0.24 [0.21 to 0.31]	0.26 [0.21 to 0.32]	0.21 [0.20 to 0.23]	0.028
eGFR (ml min^−1^)	124 [107 to 136]	127 [104 to 135]	122 [119 to 142]	0.500
Urea, (mmol l^−1^)	4.4 [3.2 to 5.3]	4.2 [2.7 to 5.3]	3.9 [3.8 to 5.3]	0.579
**Intraoperative variables**				
Baseline FiO_2_	0.42 [0.39 to 0.60]	0.40 [0.39 to 0.59]	0.48 [0.40 to 0.60]	0.363
FiO_2_ set on ventilator*	0.50 [0.40 to 0.61]	0.50 [0.40 to 0.61]	0.51 [0.40 to 0.79]	0.765
FiO_2_ during CPB	0.54 [0.50 to 0.56]	0.54 [0.50 to 0.56]	0.54 [0.50 to 0.62]	0.559
Baseline PaO_2_ (mmHg)	166 [122 to 243]	163 [121 to 246]	166 [126 to 194]	0.887
Minimum intraoperative PaO_2_ (mmHg)	89 [62 to 115]	90 [61 to 122]	82 [63 to 89]	0.380
Maximum intraoperative PaO_2_ (mmHg)	212 [183 to 253]	209 [176 to 252]	231 [193 to 276]	0.446
Baseline SpO_2_ (%)	98 [97 to 100]	98 [97 to 100]	99 [98 to 100]	0.445
Baseline SrO_2_ (%)	76 ± 7	77 ± 7	75 ± 4	0.213
Baseline ScO_2_ (%)	66 ± 8	65 ± 7	68 ± 9	0.437
Baseline SrO_2_-ScO_2_ gradient (%)	11 ± 7	12 ± 7	8 ± 7	0.218
Hypotension, time below threshold (min)	60 [36 to 95]	54 [35 to 86]	81 [68 to 100]	0.091
Hypotension, area under the threshold (mmHg min)	405 [188 to 667]	332 [185 to 662]	475 [441 to 789]	0.162
Hypotension, time-weighted average (mmHg)	2.26 [1.13 to 3.44]	2.12 [1.04 to 3.37]	2.91 [2.08 to 4.17]	0.438
Vasopressors and/or inotropes infusion (*n*)	41 (84%)	33 (83%)	8 (89%)	1.000
Milrinone	37 (76%)	29 (73%)	8 (89%)	0.420
Norepinephrine	28 (57%)	23 (58%)	5 (56%)	1.000
Epinephrine	3 (6%)	3 (8%)	0	1.000
Maximum vasoactive-inotropic score	9.2 [3.0 to 13.7]	9.3 [2.9 to 13.7]	8.9 [3.1 to 10.6]	1.000
Surgery time (min)	172 [147 to 205]	170 [151 to 195]	189 [147 to 248]	0.273
CPB time (min)	77 [62 to 89]	77 [59 to 86]	83 [68 to 121]	0.273
Aortic clamp time (min)	44 [28 to 59]	41 [27 to 57]	54 [44 to 86]	0.178
Urine output (ml kg^−1^ h^−1^)	1.0 [0.5 to 1.6]	1.0 [0.5 to 1.9]	1.2 [0.8 to 1.4]	0.786
Fluid balance (ml)	356 ± 234	348 ± 237	389 ± 233	0.644
Red blood cell transfusion (ml kg^−1^)	17 [0 to 40]	17 [3 to 37]	38 [0 to 45]	0.310
Plasma transfusion (ml kg^−1^)	9 [0 to 18]	9 [0 to 16]	8 [0 to 24]	0.896
Maximum blood lactate (mmol l^−1^)	1.5 [1.1 to 2.0]	1.5 [1.1 to 1.9]	1.8 [1.4 to 2.5]	0.195
pH	7.34 ± 0.05	7.34 ± 0.05	7.35 ± 0.06	0.641
Hemoglobin (g dl^−1^)	9.5 ± 0.9	9.5 ± 0.9	9.4 ± 0.7	0.845
**Postoperative variables and outcomes**				
Peak serum creatinine (mg dl^−1^)	0.32 [0.27 to 0.37]	0.29 [0.26 to 0.36]	0.37 [0.34 to 0.37]	0.109
Urine output (ml kg^−1^ h^−1^)	2.7 [2.0 to 3.5]	2.6 [2.0 to 3.5]	3.0 [2.7 to 3.2]	0.278
Furosemide use (*n*)	31 (63%)	24 (60%)	7 (78%)	0.455
Spironolactone use (*n*)	20 (41%)	15 (38%)	5 (56%)	0.456
ICU length of stay (days)	1 [1 to 3]	1 [1 to 3]	2 [1 to 12]	0.314
Hospital length of stay (days)	7 [7 to 10]	7 [7 to 9]	7 [7 to 21]	0.414
Postoperative complications (*n*)	24 (49%)	17 (43%)	7 (78%)	0.074
Respiratory complications (*n*)	18 (37%)	13 (33%)	5 (56%)	0.259
Infective complications (*n*)	16 (33%)	11 (28%)	5 (56%)	0.130
Cardiac complications (*n*)	11 (22%)	8 (20%)	3 (33%)	0.400
Resternotomy (*n*)	1 (2%)	0	1 (11%)	0.184
In-hospital mortality (*n*)	1 (2%)	0	1 (11%)	0.184

AKI, acute kidney injury; BMI, body mass index; BSA, body surface area; eGFR, estimated glomerular filtration rate; SrO_2_, renal tissue oxygen saturation; ScO_2_, cerebral tissue oxygen saturation; FiO_2_, fraction of inspired oxygen; PaO_2_, partial pressure of arterial oxygen; SpO_2_, peripheral oxygen saturation; CPB, cardiopulmonary bypass; ICU, intensive care unit. Data are presented as median [interquartile range] or mean ± standard deviation. *Excluding CPB

### Acute kidney injury

The incidence of AKI at the third postoperative day was 18.4%, with eight patients developing KDIGO stage 1 AKI and one patient developing stage 2 AKI. AKI developed within 24 h after surgery in six patients, on the first postoperative day in one patient, and on the second postoperative day in two patients. The diagnosis of AKI was always based on an increase in serum creatinine since the urine output criteria were never met in any patient. The use of postoperative diuretics is reported in Table 1.

### Primary outcome

**Fig. 3:**
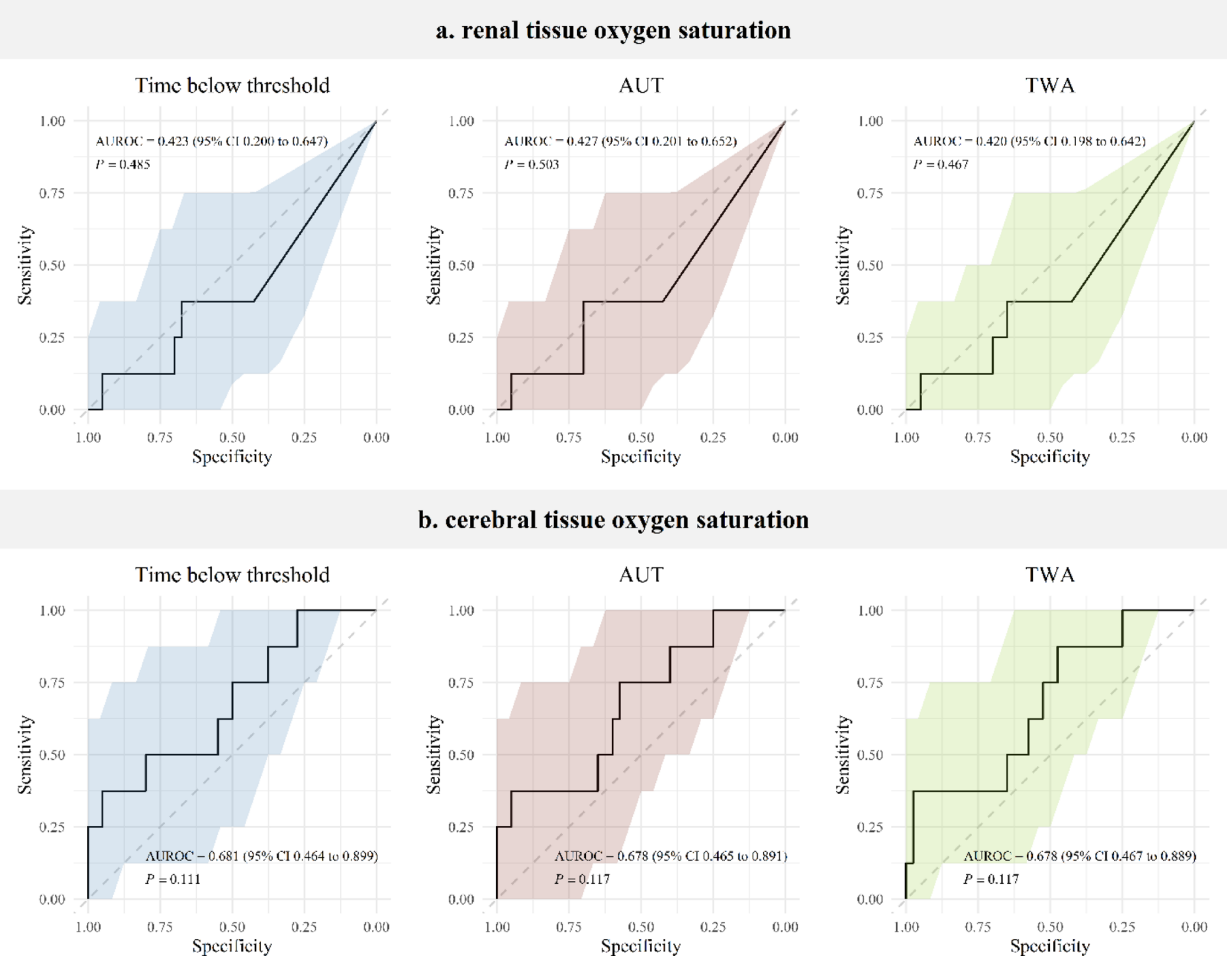
ROC curves for SrO_2_ (**a**) and ScO_2_ (**b**) relative decreases below 10% from baseline. The shaded areas represent the ROC curve 95% CI. AUT, the area under the threshold; TWA, the time-weighted average; AUROC, the area under the ROC curve

The total intraoperative time below the threshold, AUT, and TWA for SrO_2_ relative decreases below 10% and 20% from baseline did not differ between patients who developed AKI and those who did not (Table 2). Relative decreases in SrO_2_ below 20% from baseline were observed in six patients in the non-AKI group and in none of the patients in the AKI group. The total intraoperative time below the threshold, AUT, and TWA for ScO_2_ relative decreases below 10% and 20% from baseline did not differ between patients who developed AKI and those who did not (Table 2). ROC curve analysis produced non-significant AUROCs for both SrO_2_ and ScO_2_ relative decreases below baseline, irrespective of the threshold considered (Fig. 3, Online Resource 2). No correlation was found between the decreases in SrO_2_ and ScO_2_. No difference in the decrease of SrO_2_ and ScO_2_ was found when only CPB or aortic clamp times were considered (Online Resource 3).

**Table 2:** Relative intraoperative decreases in renal and cerebral tissue oxygen saturation

		10% decrease from baseline				20% decrease from baseline			
		All patients (*n* = 49)	No AKI (*n* = 40)	AKI (*n* = 9)	*P* value	All patients (*n* = 49)	No AKI (*n* = 40)	AKI (*n* = 9)	*P* value
Renal tissue oxygen saturation	Time below threshold (min)	0.1 [0 to 3.7]	0.2 [0 to 4.2]	0 [0 to 2.7]	0.485	0 [0 to 0]	0 [0 to 0]	0 [0 to 0]	0.258
	Area under the threshold (% min)	0.1 [0 to 4.9]	0.3 [0 to 5.4]	0 [0 to 2.5]	0.503	0 [0 to 0]	0 [0 to 0]	0 [0 to 0]	0.258
	Time-weighted average (%)	0 [0 to 0.03]	0 [0 to 0.03]	0 [0 to 0.01]	0.467	0 [0 to 0]	0 [0 to 0]	0 [0 to 0]	0.258
Cerebral tissue oxygen saturation	Time below threshold (min)	6 [0.7 to 26.8]	5.3 [0.4 to 22.6]	17.3 [4.8 to 92.5]	0.111	0.1 [0 to 3.6]	0 [0 to 3.4]	1.4 [0 to 16.9]	0.282
	Area under the threshold (% min)	10.4 [1.1 to 113]	9.6 [0.6 to 92.8]	36.8 [11.8 to 419.9]	0.117	0 [0 to 7.8]	0 [0 to 5.8]	2.9 [0 to 42.4]	0.323
	Time-weighted average (%)	0.08 [0.01 to 0.7]	0.07 [0 to 0.62]	0.16 [0.08 to 2.18]	0.117	0 [0 to 0.04]	0 [0 to 0]	0 [0 to 0.2]	0.323

Relative decreases below 10% or 20% from baseline renal and cerebral tissue oxygen saturation values measured during the entire surgical phase. AKI, acute kidney injury. Data are presented as median [interquartile range]

### Logistic regression analysis and secondary outcomes

In the univariate logistic regression analysis (Table 3), preoperative serum creatinine was associated with postoperative AKI. In multivariate analysis, variables significantly associated with postoperative AKI were preoperative serum creatinine (aOR = 0.71 for a 0.01 mg dl^−1^ change, 95% CI 0.50 to 0.88, *P* = 0.016), BMI (aOR = 0.29, 95% CI 0.09 to 0.66, *P* = 0.011), hypotension defined as intraoperative time with MAP < 5th percentile for age (aOR = 1.21 for a 5-minute change, 95% CI 1.05 to 1.49, *P* = 0.021), and maximum intraoperative blood lactate (aOR = 1.25 for a 0.1 mmol l^−1^ change, 95% CI 1.05 to 1.64, *P* = 0.047). Age, CPB time, maximum vasoactive-inotropic score, red blood cell transfusion, fluid balance, and relative decreases in SrO_2_ below 10% from baseline (correcting for kidney depth, included in the regression as a potential confounder) were not associated with postoperative AKI. A borderline association was found between ScO_2_ decreases below 10% from baseline and postoperative AKI in the univariate analysis (aOR = 1.04 for a 10 % min change in AUT, 95% CI 1.01 to 1.09, *P* = 0.050), reflecting a trend towards a greater intraoperative ScO_2_ reduction in patients with AKI (Table 2). However, this association was lost in the multivariate analysis.

### Post-hoc analysis

The baseline SrO_2_-ScO_2_ gradient did not differ between patients with and without AKI (Table 1). Similarly, both the cumulative and the average intraoperative SrO_2_-ScO_2_ gradients were comparable in both groups (cumulative gradient: 2138 [1307–4783] % min in patients with AKI vs. 1937 [1286–2721] % min in those without, *P* = 0.406; average gradient: 12.5 [7.4–17.3] % vs. 12.1 [8.7–14.9] %, respectively, *P* = 0.577).

**Table 3:** Logistic regression analysis

	Univariate analysis		Multivariate analysis	
	Odds ratio (95% CI)	*P* value	Adjusted odds ratio (95% CI)	*P* value
** Patient characteristics**				
Age	0.97 (0.89 to 1.01)	0.234	-	-
Body mass index	0.72 (0.42 to 1.15)	0.187	0.29 (0.09 to 0.66)	0.011
**Preoperative variables**				
Serum creatinine	0.81 (0.65 to 0.95)*	0.029	0.71 (0.50 to 0.88)*	0.016
**Intraoperative variables**				
Cardiopulmonary bypass time	1.07 (0.97 to 1.19)	0.169	-	-
SrO_2_ decrease below 10% from baseline (area under the threshold)	0.98 (0.92 to 1.05)^§^	0.758	-	-
ScO_2_ decrease below 10% from baseline (area under the threshold)	1.04 (1.01 to 1.09)^§^	0.050	-	-
Time with MAP < 5th percentile for age	1.07 (0.98 to 1.18)^†^	0.122	1.21 (1.05 to 1.49)^†^	0.021
Vasoactive-inotropic score	1.01 (0.91 to 1.11)	0.828	-	-
Red blood cell transfusion	1.01 (0.98 to 1.04)	0.433	-	-
Fluid balance	1.01 (0.98 to 1.04)	0.633	-	-
Maximum blood lactate	1.09 (0.99 to 1.21)^‡^	0.093	1.25 (1.05 to 1.64)^‡^	0.047

The McFadden’s pseudo-R-squared assessing the goodness of fit of the final logistic regression model was 0.51. SrO_2_, renal tissue oxygen saturation; ScO_2_, cerebral tissue oxygen saturation; MAP, mean arterial pressure. *For a 0.01 mg dl^−1^ change in serum creatinine. §For a 10 % min change in AUT. †For a 5-minute change in time. ‡For a 0.1 mmol l^−1^ change in blood lactate

## Discussion

In a population of forty-nine pediatric patients undergoing cardiac surgery for a congenital heart defect with a left-to-right shunt, no association was found between intraoperative SrO_2_ and ScO_2_ decreases and postoperative AKI.

The incidence of AKI in our study was 18.4%, substantially lower than expected. Previous studies conducted in pediatric patients undergoing cardiac surgery and using the KDIGO criteria for AKI diagnosis found an incidence ranging from 35 to 86% [2, 22–24]. We expected an AKI incidence on the lower end of the range (i.e., around 30%) since most previous studies included patients with both cyanotic (a strong risk factor for AKI) [5, 25] and non-cyanotic heart disease [2, 22, 23]. However, the incidence in our population of non-cyanotic patients was even lower: likely, the lower clinical and surgical complexity compared to patients included in previous studies contributed to this discrepancy. Another explanation might be the use of diuretics in the postoperative phase: the urine output criteria for AKI diagnosis according to the KDIGO definition were never met, and we cannot exclude the possibility that the use of diuretics might have masked some cases of AKI.

We found no association between intraoperative SrO_2_ decreases and postoperative AKI, as already reported by several previous studies conducted either in cohorts of only cyanotic patients [18, 24, 35], or in mixed cohorts of cyanotic and non-cyanotic patients [2, 31]. On the other hand, some studies found an association between renal tissue oxygen desaturation and postoperative AKI, even though in heterogeneous populations of cyanotic and non-cyanotic children [1, 17, 22, 26], or using other diagnostic criteria for AKI [14, 15]. Our findings differ from one of the largest studies which reported an association between intraoperative SrO_2_ decreases and postoperative AKI in 242 non-cyanotic infants undergoing cardiac surgery [16]. The authors found a 31% incidence of AKI and a 16% incidence of renal tissue desaturation below 20% from baseline, which was associated with increased odds of developing AKI. The higher AKI incidence compared with our study could be partially explained by the younger age and lower weight of the patients, while surgery and CPB times were comparable. Their population consisted exclusively of children with an isolated ventricular septal defect, which makes the results less generalisable than those of our study, which also included patients with atrial septal defects and combined defects.

The monitoring method of renal tissue oxygen saturation differed from that of previous studies in several respects. While most investigators used a single renal NIRS sensor [2, 14–18, 22], we monitored SrO_2_ bilaterally to maximize the detection of renal tissue oxygen desaturation. We ensured the proper positioning of renal NIRS sensors with ultrasound guidance and included the depth of the kidney capsule beneath the sensor as a potential confounder in the data analysis. The ultrasound location of the kidney, which has been only sporadically reported in previous studies [16, 26, 31] allowed us to ensure that the oximetry device interrogated kidney tissue (and not the overlying tissues). The penetration depth of near-infrared light approximately corresponds to half the distance between the emitting optodes and the detector, which in the case of the O3 NIRS sensors (both neonatal and pediatric) is 3 cm and 4 cm, respectively. In our patients, kidney depth was adequate (≤ 2 cm in most subjects, and > 2.5 cm only in one patient) to be reached by near-infrared light. To our knowledge, this is the first study to use the O3 Regional Oximetry for SrO_2_ monitoring in pediatric surgical patients. It is worth noting that the O3 Regional Oximetry has limitations in renal tissue oxygen saturation monitoring in healthy adults [36], while it has been successfully validated for cerebral tissue oxygen saturation monitoring in children undergoing cardiac surgery [37]. However, validation study for renal tissue oxygen saturation monitoring in children is still lacking. A single study found a good correlation between SrO_2_ and renal vein saturation in children weighing < 10 kg with heart disease, albeit with another NIRS device [11]. Nonetheless, in the absence of formal validation of the monitoring technique in this patient population, and given the wide range of ages and weights of the patients included, our SrO_2_ data and findings should be considered preliminary.

We found no association between intraoperative ScO_2_ decreases and postoperative AKI. This finding is in line with the literature, since most studies conducted in children undergoing cardiac surgery found no association [1, 14, 18, 35], whereas only one study found lower intraoperative ScO_2_ values in patients developing AKI [17]. A trend towards greater ScO_2_ decreases below 10% from baseline could be observed, but did not meet statistical significance especially when ROC curves and multivariate logistic regression analysis were considered. Since the actual power of the study was lower than planned due to the low incidence of AKI, the significance of this finding remains to be clarified.

All patients underwent surgery with CPB, and the adequacy of organ perfusion was also monitored by assessing intraoperative variables, such as urine output and blood lactate levels. This targeted hemodynamic approach might partially mask any relationship between tissue oxygen desaturation and AKI. The association between intraoperative blood lactate and postoperative AKI suggests that, in our study, blood lactate was a better marker of hypoperfusion compared with renal and cerebral tissue oxygen saturation. This finding is consistent with previous studies that have shown an association between elevated postoperative blood lactate and AKI in pediatric cardiac surgery [25, 38]. One study showed that a reduction in the SrO_2_-ScO_2_ gradient was associated with an increase in lactate during neonatal CPB [30], supporting a role for a reduced SrO_2_-ScO_2_ gradient as a marker of hypoperfusion. We conducted a post-hoc analysis to investigate whether patients with AKI exhibited a reduced intraoperative SrO_2_-ScO_2_ gradient, but found no difference compared to patients without AKI. This analysis should be considered exploratory, as it was not a prespecified outcome of our study.

Other variables associated with postoperative AKI were preoperative serum creatinine values, BMI, and intraoperative hypotension defined as the time with MAP < 5th percentile for age. Low preoperative serum creatinine is a known risk factor for postoperative AKI after pediatric cardiac surgery [5, 25, 39], and might be a proxy for a younger age, another well-established risk factor [4, 40, 41]. As suggested by other authors [42], this finding might also be explained by the KDIGO definition of AKI itself: the lower the baseline serum creatinine, the lower the absolute increase required to meet the criteria for AKI stage 1 (×1.5–1.9 baseline serum creatinine). Intraoperative hypotension has been demonstrated to be a risk factor for AKI in adults undergoing cardiac surgery [43, 44], whereas it has not yet been thoroughly investigated in pediatric cardiac surgery. One study found that 88% of children developing AKI had intraoperative hypotension, but no comparison was made with children without AKI [4]. Other studies showed an association between intraoperative blood pressure variability and postoperative AKI [45], and between low MAP values during CPB and intraoperative kidney injury [9]. Since the association between hypotension and AKI was a secondary outcome of our study, this finding needs to be confirmed in other settings.

This study has several limitations. First, the incidence of postoperative AKI was lower than expected, so the study is underpowered for the primary outcome. Second, the O3 Regional Oximetry has still not been validated for SrO_2_ monitoring in children. Third, we investigated only relative decreases of SrO_2_ and ScO_2_ below two arbitrary thresholds (below 10% and 20% from baseline), even though such thresholds were used in other studies [16, 31, 32]. It can be debated whether other definitions or thresholds can better assess renal and cerebral tissue oxygen desaturation. Fourth, the attending anesthetists were not blinded to SrO_2_ and ScO_2_ values, and abnormal tissue oxygen saturation values could have influenced the intraoperative respiratory and hemodynamic management. Finally, baseline SrO_2_ and ScO_2_ values were obtained after induction of anesthesia. While this may not represent a true ‘physiological’ baseline, due to factors such as reduced cerebral and systemic oxygen consumption and hyperoxemia during mechanical ventilation, we believe it stands as a clinically relevant reference point. In pediatric cardiac surgery, measuring clinical variables under general anesthesia or sedation is closely aligned with clinical practice, especially in the intraoperative and immediate postoperative phase. Moreover, this method ensures standardized baseline conditions across patients, and has already been employed by other authors [16, 31, 32]. Therefore, this approach can reasonably provide meaningful baseline values for assessing the subsequent effects of surgery, CPB, and other intraoperative events affecting regional oxygen saturation.

In conclusion, a decrease in intraoperative SrO_2_ and ScO_2_ values was not associated with the development of postoperative AKI in pediatric patients undergoing corrective surgery for non-cyanotic congenital heart disease with a left-to-right shunt, performed with CPB. Preoperative serum creatinine, BMI, intraoperative blood lactate, and intraoperative hypotension, defined as the time with MAP < 5th percentile for age, were associated with the development of postoperative AKI.

## Supplementary Information

Below is the link to the electronic supplementary material.

Supplementary Material 1

Supplementary Material 2

## Data Availability

No datasets were generated or analysed during the current study.
