# Supplementary material for: Intraoperative renal and cerebral tissue oxygen saturation measurements to predict postoperative acute kidney injury in pediatric cardiac surgery: a prospective observational study
Source: J Clin Monit Comput. 2025 Sep 3;40(2):405–16. doi: 10.1007/s10877-025-01345-4 (PMC13053570; doi:10.1007/s10877-025-01345-4)
Supplement: Supplementary file 1 — Supplementary Material 1 [file 10877_2025_1345_MOESM1_ESM.docx]

**Online Resource 1** Intraoperative FiO_2_

|  | **10% decrease from baseline** | | | | | | **20% decrease from baseline** | | | | | |
| --- | --- | --- | --- | --- | --- | --- | --- | --- | --- | --- | --- | --- |
|  | **Renal tissue oxygen saturation** | | | **Cerebral tissue oxygen saturation** | | | **Renal tissue oxygen saturation** | | | **Cerebral tissue oxygen saturation** | | |
|  | Below  threshold | Above  threshold | *P* value | Below  threshold | Above  threshold | *P* value | Below  threshold | Above  threshold | *P* value | Below  threshold | Above  threshold | *P* value |
| **FiO_2_ set on ventilator*** | 0.60  [0.44 to 0.97] | 0.60  [0.40 to 0.90] | 0.986 | 0.58  [0.40 to 0.80] | 0.60  [0.40 to 0.81] | 0.803 | - | - | - | 0.57  [0.42 to 0.63] | 0.56  [0.43 to 0.80] | 0.832 |
| **FiO_2_ during CPB** | 0.55  [0.50 to 0.61] | 0.55  [0.53 to 0.60] | 0.849 | 0.54  [0.50 to 0.60] | 0.50  [0.50 to 0.57] | 0.247 | 0.58  [0.54 to 0.62] | 0.59  [0.53 to 0.67] | 1 | 0.54  [0.48 to 0.65] | 0.51  [0.50 to 0.56] | 0.512 |

Comparison of FiO_2_ values between intraoperative sections with SrO_2_ and ScO_2_ values respectively below and above threshold. Since SrO_2_ decreases below 20% from baseline occurred in a minority of patients, and mostly during CPB, not enough data were available to calculate the FiO_2_ set on ventilator, which is not reported. SrO_2_, renal tissue oxygen saturation; ScO_2_, cerebral tissue oxygen saturation; FiO_2_, fraction of inspired oxygen; CPB, cardiopulmonary bypass. Data are presented as median [interquartile range]. *Excluding CPB.

**Online Resource 2** ROC curve analysis

|  |  | **10% decrease from baseline** | | **20% decrease from baseline** | |
| --- | --- | --- | --- | --- | --- |
|  |  | **AUROC (95% CI)** | ***P* value** | **AUROC (95% CI)** | ***P* value** |
| **Renal tissue oxygen saturation** | **Time below the threshold** (min) | 0.423 (0.200 to 0.647) | 0.485 | 0.425 (0.369 to 0.481) | 0.258 |
|  | **Area under the threshold** (% min) | 0.427 (0.201 to 0.652) | 0.503 | 0.425 (0.369 to 0.481) | 0.258 |
|  | **Time-weighted average** (%) | 0.420 (0.198 to 0.642) | 0.467 | 0.425 (0.369 to 0.481) | 0.258 |
| **Cerebral tissue oxygen saturation** | **Time below the threshold** (min) | 0.681 (0.464 to 0.899) | 0.111 | 0.617 (0.375 to 0.860) | 0.282 |
|  | **Area under the threshold** (% min) | 0.678 (0.465 to 0.891) | 0.117 | 0.608 (0.368 to 0.847) | 0.323 |
|  | **Time-weighted average** (%) | 0.678 (0.467 to 0.889) | 0.117 | 0.608 (0.370 to 0.846) | 0.323 |

AUROC, area under the Receiver Operating Characteristic curve.

**Online Resource 3** Relative decreases in renal and cerebral tissue oxygen saturation during cardiopulmonary bypass and aortic clamp

|  |  |  | **10% decrease from baseline** | | | | **20% decrease from baseline** | | | |
| --- | --- | --- | --- | --- | --- | --- | --- | --- | --- | --- |
|  |  |  | **All patients**  **(*n*=49)** | **No AKI**  **(*n*=40)** | **AKI**  **(*n*=9)** | ***P* value** | **All patients**  **(*n*=49)** | **No AKI**  **(*n*=40)** | **AKI**  **(*n*=9)** | ***P* value** |
| **Renal tissue oxygen saturation** | **CPB** | **Time below the threshold** (min) | 0 [0 to 2.9] | 0 [0 to 3.7] | 0 [0 to 1.4] | 0.781 | 0 [0 to 0] | 0 [0 to 0] | 0 [0 to 0] | 0.309 |
|  |  | **Area under the threshold** (% min) | 0 [0 to 2.3] | 0 [0 to 4.9] | 0 [0 to 1.3] | 0.829 | 0 [0 to 0] | 0 [0 to 0] | 0 [0 to 0] | 0.309 |
|  |  | **Time-weighted average** (%) | 0 [0 to 0.04] | 0 [0 to 0.08] | 0 [0 to 0.02] | 0.805 | 0 [0 to 0] | 0 [0 to 0] | 0 [0 to 0] | 0.309 |
|  | **Aortic clamp** | **Time below the threshold** (min) | 0 [0 to 0.7] | 0 [0 to 0.7] | 0 [0 to 0.3] | 0.705 | 0 [0 to 0] | 0 [0 to 0] | 0 [0 to 0] | 0.448 |
|  |  | **Area under the threshold** (% min) | 0 [0 to 0.7] | 0 [0 to 0.8] | 0 [0 to 0.3] | 0.680 | 0 [0 to 0] | 0 [0 to 0] | 0 [0 to 0] | 0.448 |
|  |  | **Time-weighted average** (%) | 0 [0 to 0.01] | 0 [0 to 0.02] | 0 [0 to 0] | 0.633 | 0 [0 to 0] | 0 [0 to 0] | 0 [0 to 0] | 0.448 |
| **Cerebral tissue oxygen saturation** | **CPB** | **Time below the threshold** (min) | 4.1 [0 to 18.3] | 3.7 [0 to 14.2] | 13.2 [0.4 to 49.9] | 0.269 | 0 [0 to 1.2] | 0 [0 to 0.8] | 0 [0 to 10.4] | 0.650 |
|  |  | **Area under the threshold** (% min) | 7.3 [0 to 49.4] | 6.2 [0 to 44.3] | 26.8 [0.4 to 233.5] | 0.334 | 0 [0 to 2.2] | 0 [0 to 1.1] | 0 [0 to 25.5] | 0.672 |
|  |  | **Time-weighted average** (%) | 0.12 [0 to 0.52] | 0.1 [0 to 0.49] | 0.25 [0.01 to 2.66] | 0.393 | 0 [0 to 0.04] | 0 [0 to 0] | 0 [0 to 0.3] | 0.650 |
|  | **Aortic clamp** | **Time below the threshold** (min) | 0.5 [0 to 7.9] | 0.2 [0 to 5.5] | 9.1 [0 to 31.6] | 0.189 | 0 [0 to 0.1] | 0 [0 to 0] | 0 [0 to 7.9] | 0.236 |
|  |  | **Area under the threshold** (% min) | 0.3 [0 to 26.2] | 0.2 [0 to 11] | 20.4 [0 to 151.1] | 0.219 | 0 [0 to 0] | 0 [0 to 0] | 0 [0 to 14.3] | 0.236 |
|  |  | **Time-weighted average** (%) | 0.01 [0 to 0.74] | 0.01 [0 to 0.45] | 0.26 [0 to 3.05] | 0.242 | 0 [0 to 0] | 0 [0 to 0] | 0 [0 to 0.3] | 0.251 |

Relative decreases in renal and cerebral tissue oxygen saturation below 10% or 20% from baseline values measured during cardiopulmonary bypass (CPB) and during aortic clamp. AKI, acute kidney injury. Data are presented as median [interquartile range].
