# Supplementary material for: Intraoperative renal and cerebral tissue oxygen saturation measurements to predict postoperative acute kidney injury in pediatric cardiac surgery: a prospective observational study
Source: J Clin Monit Comput. 2025 Sep 3;40(2):405–16. doi: 10.1007/s10877-025-01345-4 (PMC13053570; doi:10.1007/s10877-025-01345-4)
Supplement: Supplementary file 2 — Supplementary Material 2 [file 10877_2025_1345_MOESM2_ESM.docx]

**STROBE Statement**

|  | Item No. | Recommendation | Page  No. | Relevant text from manuscript |
| --- | --- | --- | --- | --- |
| **Title and abstract** | 1 | (*a*) Indicate the study’s design with a commonly used term in the title or the abstract | 1, 2 | a prospective observational study |
|  |  | (*b*) Provide in the abstract an informative and balanced summary of what was done and what was found | 2 |  |
| Introduction | | | |  |
| Background/rationale | 2 | Explain the scientific background and rationale for the investigation being reported | 3 | One key pathogenetic mechanism involved in the development of AKI is perioperative renal hypoperfusion and hypoxia |
| Objectives | 3 | State specific objectives, including any prespecified hypotheses | 3 | We designed this study to hypothesize that a decrease in intraoperative SrO2 and ScO2 values is associated with the development of postoperative AKI in pediatric patients undergoing corrective surgery for non-cyanotic congenital heart disease with a left-to-right shunt |
| Methods | | | |  |
| Study design | 4 | Present key elements of study design early in the paper | 4 |  |
| Setting | 5 | Describe the setting, locations, and relevant dates, including periods of recruitment, exposure, follow-up, and data collection | 4 | This prospective observational study was conducted at the University Medical Center Groningen, the Netherlands, from 2021 to 2024 |
| Participants | 6 | (*a*) *Cohort study*—Give the eligibility criteria, and the sources and methods of selection of participants. Describe methods of follow-up  *Case-control study*—Give the eligibility criteria, and the sources and methods of case ascertainment and control selection. Give the rationale for the choice of cases and controls  *Cross-sectional study*—Give the eligibility criteria, and the sources and methods of selection of participants | 4 | We included patients under eighteen years of age with a non-cyanotic congenital heart defect with a left-to-right shunt scheduled for corrective cardiac surgery with CPB. The exclusion criteria were […] |
|  |  | (*b*) *Cohort study*—For matched studies, give matching criteria and number of exposed and unexposed  *Case-control study*—For matched studies, give matching criteria and the number of controls per case |  |  |
| Variables | 7 | Clearly define all outcomes, exposures, predictors, potential confounders, and effect modifiers. Give diagnostic criteria, if applicable | 6 | Postoperative AKI occurring within the third postoperative day was defined according to the ‘Kidney Disease: Improving Global Outcomes’ (KDIGO) criteria. […] The primary outcome of the study was the association between intraoperative decreases in SrO2 and ScO2 values, and postoperative AKI |
| Data sources/ measurement | 8* | For each variable of interest, give sources of data and details of methods of assessment (measurement). Describe comparability of assessment methods if there is more than one group | 5 | Two O3® Regional Oximetry sensors connected to a Root® oximetry monitor […] were placed bilaterally upon the forehead for ScO_2_ monitoring.  SrO_2_ and ScO_2_ values […] were measured continuously and automatically recorded in our electronic patient high-frequency measurements database |
| Bias | 9 | Describe any efforts to address potential sources of bias | 5 | ultrasound guidance was used to locate the kidneys, and the O3 NIRS sensors were placed on the overlying skin region. The depth of the kidney capsule beneath the skin surface was recorded |
| Study size | 10 | Explain how the study size was arrived at | 4 | Previous studies conducted in pediatric cardiac surgery and using the KDIGO criteria found an AKI incidence ranging from 35% to 86% [2, 22–24]. These studies included both cyanotic and non-cyanotic patients. Since cyanotic disease is a known risk factor for AKI [5, 25] we expected the AKI incidence in non-cyanotic patients to be on the lower side; therefore, we assumed a 30% AKI incidence to calculate the sample size. To obtain an Area Under the Receiver Operating Characteristic curve (AUROC) of 0.75 for predicting AKI [15, 26], with 80% power and a two-tailed 95% confidence level, a sample size of 46 patients was required. This was increased to 50 to compensate for possible missing data. |

| Quantitative variables | 11 | Explain how quantitative variables were handled in the analyses. If applicable, describe which groupings were chosen and why | 7 | For both SrO_2_ and ScO_2_, we calculated the total intraoperative time below the threshold, the area under the threshold (AUT), and the time-weighted average |
| --- | --- | --- | --- | --- |
| Statistical methods | 12 | (*a*) Describe all statistical methods, including those used to control for confounding | 6, 7 |  |
|  |  | (*b*) Describe any methods used to examine subgroups and interactions | 7 | the relationship between known or potential predictors of AKI and postoperative AKI was assessed using a logistic regression model |
|  |  | (*c*) Explain how missing data were addressed | 7 | Linear interpolation for SrO_2_, ScO_2_ and MAP values was used for intraoperative sections of missing data shorter than 5 minutes. If the section was longer than 5 minutes, the entire data series was omitted from the analysis. No inference was made for other missing data |
|  |  | (*d*) *Cohort study*—If applicable, explain how loss to follow-up was addressed  *Case-control study*—If applicable, explain how matching of cases and controls was addressed  *Cross-sectional study*—If applicable, describe analytical methods taking account of sampling strategy | Not applicable |  |
|  |  | (*e*) Describe any sensitivity analyses |  |  |
| Results | | | | |
| Participants | 13* | (a) Report numbers of individuals at each stage of study—eg numbers potentially eligible, examined for eligibility, confirmed eligible, included in the study, completing follow-up, and analysed | 9 |  |
|  |  | (b) Give reasons for non-participation at each stage | 9 |  |
|  |  | (c) Consider use of a flow diagram | Figure 2 |  |
| Descriptive data | 14* | (a) Give characteristics of study participants (eg demographic, clinical, social) and information on exposures and potential confounders | Table 1 |  |
|  |  | (b) Indicate number of participants with missing data for each variable of interest | 8 |  |
|  |  | (c) *Cohort study*—Summarise follow-up time (eg, average and total amount) | Not applicable |  |
| Outcome data | 15* | *Cohort study*—Report numbers of outcome events or summary measures over time | 12 | The incidence of AKI at the third postoperative day was 18.4%, with eight patients developing KDIGO stage 1 AKI and one patient developing stage 2 AKI |
|  |  | *Case-control study—*Report numbers in each exposure category, or summary measures of exposure |  |  |
|  |  | *Cross-sectional study—*Report numbers of outcome events or summary measures |  |  |
| Main results | 16 | (*a*) Give unadjusted estimates and, if applicable, confounder-adjusted estimates and their precision (eg, 95% confidence interval). Make clear which confounders were adjusted for and why they were included | Table 3 |  |
|  |  | (*b*) Report category boundaries when continuous variables were categorized | Not applicable |  |
|  |  | (*c*) If relevant, consider translating estimates of relative risk into absolute risk for a meaningful time period | Not applicable |  |

| Other analyses | 17 | Report other analyses done—eg analyses of subgroups and interactions, and sensitivity analyses | Not applicable |  |
| --- | --- | --- | --- | --- |
| Discussion | | | | |
| Key results | 18 | Summarise key results with reference to study objectives | 17 |  |
| Limitations | 19 | Discuss limitations of the study, taking into account sources of potential bias or imprecision. Discuss both direction and magnitude of any potential bias | 19 |  |
| Interpretation | 20 | Give a cautious overall interpretation of results considering objectives, limitations, multiplicity of analyses, results from similar studies, and other relevant evidence | 19 |  |
| Generalisability | 21 | Discuss the generalisability (external validity) of the study results | 17-20 |  |
| Other information | |  | | |
| Funding | 22 | Give the source of funding and the role of the funders for the present study and, if applicable, for the original study on which the present article is based | 25 | This work was supported by the Department of Anesthesiology of the University of Groningen, University Medical Center Groningen, Groningen, the Netherlands. |

*Give information separately for cases and controls in case-control studies and, if applicable, for exposed and unexposed groups in cohort and cross-sectional studies.

**Note:** An Explanation and Elaboration article discusses each checklist item and gives methodological background and published examples of transparent reporting. The STROBE checklist is best used in conjunction with this article (freely available on the Web sites of PLoS Medicine at http://www.plosmedicine.org/, Annals of Internal Medicine at http://www.annals.org/, and Epidemiology at http://www.epidem.com/). Information on the STROBE Initiative is available at www.strobe-statement.org.
